# Rheumatologists’ expectations and perceptions of T-cell redirecting therapies: results from a cross-sectional survey

**DOI:** 10.1007/s00296-026-06300-3

**Published:** 2026-09-26

**Authors:** Anna Meinecke, Johannes Knitza, Thorben Witte, Judith Rademacher, Felix Müller, Christina Düsing, Marco Zeeck, Ricardo Grieshaber-Bouyer, Melanie Hagen, Isabell Haase, Marie-Therese Holzer

**Affiliations:** 1 Department of Rheumatology and Immunology, Hannover Medical School, Carl-Neuberg-Str. 1, 30625 Hannover, Germany; 2 PRACTIS Clinician Scientist Program, Dean’s Office for Academic Career Development, Hannover Medical School, Hannover, Germany; 3 Working Group Young Rheumatology, German Society for Rheumatology, Berlin, Germany; 4 Institute for Digital Medicine, Philipps University Marburg, Marburg, Germany; 5 Department of Rheumatology and Clinical Immunology, Charité-Universitätsmedizin Berlin, Berlin, Germany; 6 Department of Gastroenterology, Infectiology and Rheumatology (Including Nutrition Medicine), Charite Universitätsmedizin Berlin, Berlin, Germany; 7 Division of Rheumatology and Clinical Immunology, Department of Medicine 3, University Hospital Augsburg, Augsburg, Germany; 8 Department of Rheumatology, University Hospital Düsseldorf, Medical Faculty of Heinrich-Heine University, Düsseldorf, Germany; 9 Rheuma Zentrum Lübeck, Lübeck, Germany; 10 Deutsches Zentrum Immuntherapie (DZI), Friedrich-Alexander Universität (FAU) Erlangen-Nürnberg, Erlangen, Germany; 11 Department of Internal Medicine 3–Rheumatology and Immunology, Friedrich-Alexander Universität (FAU) Erlangen-Nürnberg and Universitätsklinikum Erlangen, Erlangen, Germany; 12 Division of Rheumatology and Systemic Inflammatory Diseases, III. Department of Medicine, University Medical Center Hamburg-Eppendorf, Hamburg, Germany

**Keywords:** Immunotherapy, adoptive, Surveys and questionnaires, Autoimmune diseases, Health, knowledge, attitudes, practice, Professional practice

## Abstract

T-cell redirecting therapies (TCRTs), including Chimeric antigen receptor (CAR)-T cells and T-cell engagers (TCEs), are emerging as transformative treatments for autoimmune diseases. However, clinical application is currently restricted to specialized centers, leaving most clinicians with limited first-hand experience. We aimed to assess the knowledge, perception, and barriers regarding TCRTs among German rheumatologists. A prospective, web-based survey was distributed to German rheumatologists via congresses and digital channels from September 2024 through January 2025. Drafting the design and reporting of the survey results was performed regarding international recommendations as the “checklist for reporting results of internet e-surveys” (CHERRIES). Statistical analysis was conducted with SPSS. Of 98 participants (median age 43 years) the majority was based at university hospitals (46.3%) and 94.8% were willing to refer patients to specialized centers. However, respondents considered only 5% of their patients suitable for TCRT, primarily those with severe, refractory connective tissue diseases. The main barriers cited were high costs (82%), limited clinical experience (65.5%) and applicability in only a limited number of patients (35.4%). Knowledge of side effects (e.g., cytokine release syndrome, infections, hypogammaglobulinemia) and contraindications (e.g. heart failure, chronic infections) was generally accurate. Yet, the self-appraisal of existing knowledge on TCRT was only seen as medium by the questioned rheumatologists (median of 4.86, on a 0 to 10 scale with 10 is best knowledge). Participating German rheumatologists demonstrated a high willingness to refer patients for TCRTs and appeared to possess good safety knowledge. However, the therapy is currently viewed as a niche option for highly active, refractory cases, with costs and limited data serving as primary barriers to broader implementation.

**Supplementary Information:**

The online version contains supplementary material available at https://doi.org/10.1007/s00296-026-06300-3.

## Introduction

T cell redirecting therapies (TCRTs) encompass a class of immune interventions designed to harness and retarget endogenous T cells cytotoxicity to kill target cells [1].

Two principal modalities dominate this field:

Chimeric antigen receptor (CAR) T cells are modified T cells to express a CAR transgene which allows T cells to recognize cells expressing the corresponding antigen to enable potent and sustained cytotoxic responses [2]. These therapies can be engineered ex vivo out of autologous and allogenic T cells, or in vivo (e.g. via mRNA or viral vector) [3].

In contrast, T cell engagers (TCE) are biologics containing at least two distinct binding domains. The simultaneous engagement of CD3 on the T cell surface and target cells via a specific antigen leads to T cell activation via its natural T cell receptor and depletion of the target cell via a cytolytic synapse [4].

TCRTs have been successfully used for hematooncological diseases and revolutionized the therapeutic approaches over the last few years. Initially addressing B cell targets like CD19, CD20 and BCMA [5, 6], newer TCRT trials are now targeting antigens like EGFR for solid tumors as well [7] and several TCEs targeting solid tumor antigens have already been approved [8].

Next to treating hematologic and oncologic diseases, CAR T and TCE therapies have also been applied for severe autoimmune diseases such as systemic lupus erythematosus, myasthenia gravis, systemic sclerosis, rheumatoid arthritis, Sjogren’s disease, idiopathic inflammatory myositis, Grave’s disease, IgG4-related disease, ANCA-associated vasculitis [9–13].

While the landscape of clinical trials is evolving quickly, TCRTs are currently only used in specialized centers, and the number of published patients is still low. Up to date, no licensed application of TCRT in rheumatology exists. Consequently, most clinicians have limited first-hand experience of TCRTs. Expanding patient access to this treatment requires greater awareness among referring physicians.

As first cases of both CAR T cell and TCE therapy in rheumatic autoimmune diseases were reported from Germany, most (predominantly early) clinical trials on CAR T cell therapy are conducted in China, the United States and in Europe mainly in Germany [14, 15]. Yet, TCE therapy results consist mainly of case reports and are reported by German rheumatology centers [15]. Due to the experimental state of TCRT, the perception of potentially referring rheumatologists seems especially important.

At present, however, it is uncertain to what extent rheumatologists in Germany are adequately informed about this therapeutic option and its associated opportunities and constraints. Thus, this study assessed primarily rheumatologists' knowledge and perceptions of TCRTs in autoimmune diseases and secondarily evaluated the perception of their clinical applicability, treatment-associated adverse events and contraindications, perceived barriers to implementation, and referral patterns.

## Methods

A web-based cross-sectional survey was created by the Working Group Young Rheumatology of the German Society for Rheumatology. A formal enquiry was submitted to the ethics committee of the Philipps University Marburg, Germany (24–224 ANZ). Due to the anonymous and non-interventional nature of the study, it was deemed as exempted from IRB review. Questionnaire designing, reporting of results and manuscript preparation was performed regarding the recommendations recently published as well as regarding the checklist for reporting results of internet e-surveys (CHERRIES) [16, 17].

An expert panel was built compounding of 11 members of the Young Working Group of colleagues with known expertise and clinical experience in TCRTs as well as of colleagues from hospitals and practices without direct access to TCRT. Within this expert panel, we identified the main areas of interest: (i) the therapeutic potential, (ii) the barriers TCRTs and (iii) physician knowledge regarding TCRTs.

Individual literature research on these topics was performed and a first survey drafted.

After the internal review process and pilot testing through the members of the Working Group Young Rheumatology, 17 final items were selected for the survey (supplementary material). The questions were thoroughly assessed and modified to ensure validity. Survey completion was possible within 10 min or less. The survey was prepared as a google form in German which could be accessed and answered anonymously, thus, view rate and IP-address were not gathered.

The survey was distributed at the 52nd Congress of the German Society for Rheumatology, with mobile devices (tablet) to participants and has been accessible via email, social media and QR code from 18 September 2024 until 5 January 2025. Board-certified rheumatologists and fellows in subspecialty training were eligible to participate. All participants agreed to anonymized data analyzation and publication of retrieved results.

The descriptive analysis was conducted using IBM SPSS Statistics Version 28.0.1.0 for Windows (SPSS Inc., Chicago, Illinois, USA). Categorical variables are presented as frequencies and percentages. Continuous variables are presented as mean ± standard deviation (SD) or median with interquartile range (IQR), as appropriate. To compare respondents from university and non-university hospitals, Fisher's exact test was used for dichotomous variables. For continuous variables, the distribution of the data was assessed using the Shapiro–Wilk test. For variables that were not normally distributed, the Mann–Whitney U test was applied. No imputation of missing data was performed; analyses were based on the available responses for each individual question. While certain inquiries were designated as obligatory, respondents were permitted to opt for the "no response" option, thereby abstaining from providing a response. Consequently, the number of respondents varied between individual analyses. All statistical tests were two-sided, and p-values less than 0.05 were considered statistically significant. The graphics were created with GraphPad Prism version 9.0.2 (GraphPad, San Diego, California, USA).

## Results

### Study population

Overall, 98 rheumatologists completed the survey. The mean age of the participants was 43.0 years with a female proportion of 53.1%.

32.3% of the participants were residents/fellows while 67.7% were board-certified in rheumatology (Table 1). Most participants (46.3%) worked in university hospitals and 21.1% had their own practice.

**Table 1:** Study population characteristics

*Clinical Characteristics*		
Women		52/98 (53.1%)
Age in years	Mean ± SD	43.0 ± 12.4
*Position*		
Resident		30/93 (32.3%)
Board-certificated Rheumatologist		42/93 (45.2%)
Senior consultant		14/93 (15.1%)
Chief physician		7/93 (7.5%)
*Type of workplace*		
University Hospital		44/95 (46.3%)
Maximum-care clinic		4/95 (4.2%)
Specialist clinic		6/95 (6.3%)
Primary care clinic		6/95 (6.3%)
Own practice		20/95 (21.1%)
Employed in practice or medical care center		15/95 (15.8%)
*Specific items*		
Willing to refer patients to centers		91/96 (94.8%)
Suitable Patients for TCRTs (in %)	Mean ± SD	4.66 ± 6.98
Knowledge of TCRTs (ordinary Scale from 1–10)	Mean	4.86

*SD* Standard deviation

The majority of rheumatologists (91/96, 94.8%) would refer patients in specialized centers to apply TCRT cell therapy (Table 1), with 3 additional rheumatologists stating that they can provide TCRT in their clinics themselves. However, on average participants considered only 5% of their patients suitable for TCRT (Table 1).

**Fig. 1:**
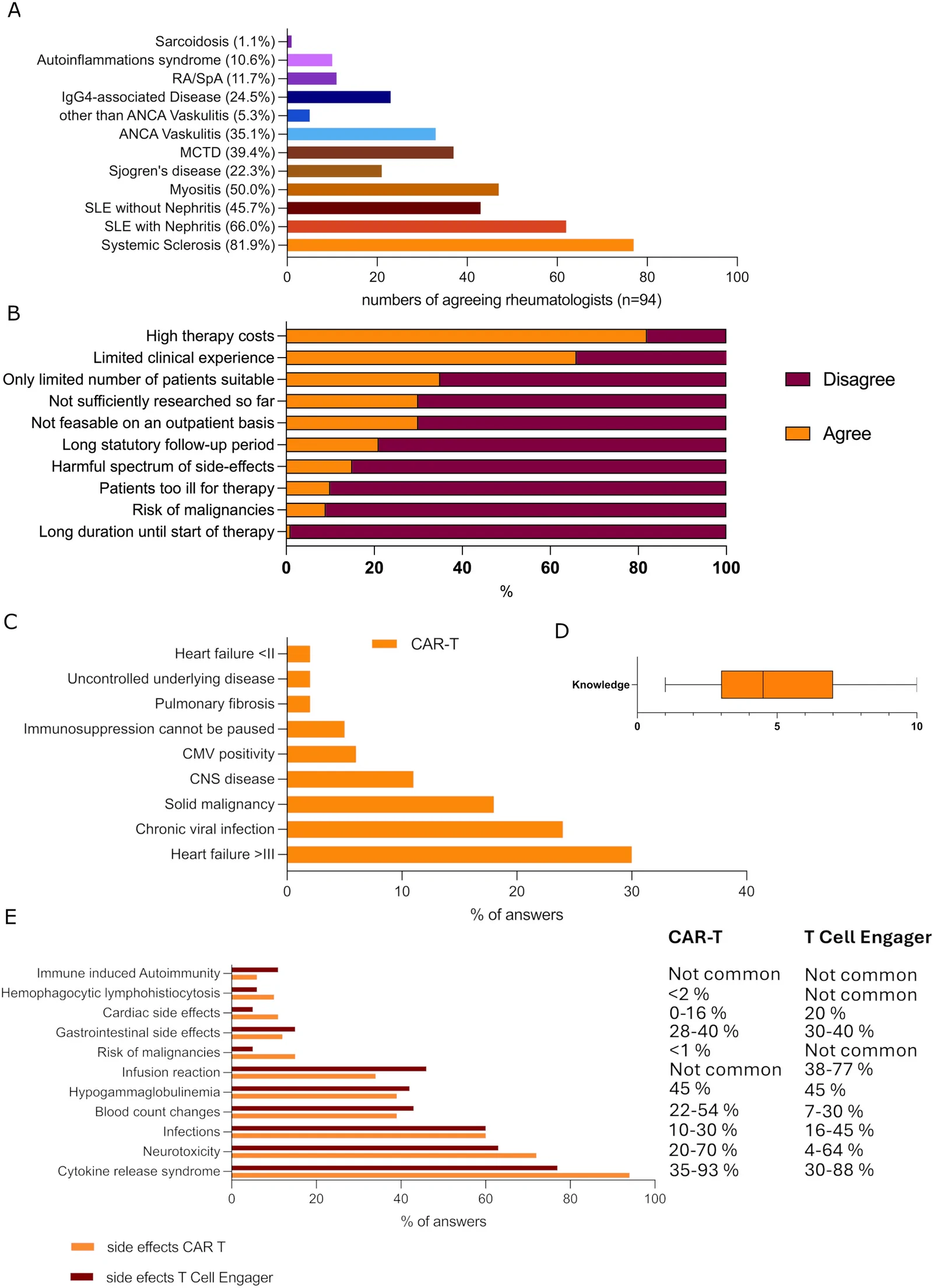
Perceptions and expectations of German Rheumatologist regarding novel T-cell redirecting therapies. **A** Suitable diseases for TCRTs ranked by rheumatologists. **A** Rheumatologists’ ranking of diseases considered suitable for T-cell redirecting therapies (TCRTs; *n* = 94). **B** Perceived barriers to the use of TCRTs (*n* = 96). **C** Rheumatologists’ ranking of contraindications to CAR-T therapy (*n* = 89). **D** Self-assessed knowledge of TCRTs, rated on a scale from 0 to 10, with a mean score of 4.86 (*n* = 98). **E** Participating rheumatologists’ ranking of potential adverse events associated with TCRTs compared with reported frequencies of adverse events following chimeric antigen receptor T-cell (CAR-T) therapy [2, 18, 35–39] and T-cell engager (TCE) therapy [21, 40–43] in the literature (*n* = 95 for CAR-T therapy and *n* = 79 for TCE therapy)

Connective tissue diseases were considered particularly suitable for TCRT: Amongst disease entities, systemic lupus erythematosus (SLE) with (62 of 94 rheumatologists, 66.0%) and without (43/94, 45.7%) lupus nephritis, myositis (47/94, 50.0%) and systemic sclerosis (77/94, 81.9%) were stated most often as candidates for TCRT (Fig. 1A). On the other hand, rheumatoid arthritis and spondyloarthritis were stated as suitable by only 11.7% (11/94).

Similarly, when participants were presented different case vignettes (Supplementary Information), significant organ involvement plus therapy refractory cases seemed to be factors favoring TCRT: CAR T cell therapy was suggested by 70.1% (69 of 98 respondents) for a systemic sclerosis patient with lung involvement and multiple previous therapies, whereas a patient with SLE with “solely” skin, joint and hair involvement was seen as not suitable by 91.8% (90/98) of the rheumatologists and an intubated patient with respiratory insufficiency with anti-neutrophil cytoplasmic antibody (ANCA)-vasculitis and only prednisolone therapy beforehand by 76.3% (75/98).

As most important barriers of TCRT, high treatment costs (indicated by 82.3% of the 98 rheumatologists), limited clinical experience (65.5%), applicability in only a limited number of patients (35.4%) as well as insufficient research and unfeasibility in an outpatient setting (30.2% respectively) were stated by the rheumatologists (Fig. 1B).

For addressing the current knowledge on TCRT, most common side effects were asked. Overall, self-appraisal of existing knowledge on TCRT was seen as moderate by most rheumatologists (median of 4.86 on a 0 to 10 scale with 10 is best knowledge) (Fig. 1D).

Most participants named cytokine release syndrome (CRS), neurotoxicity, infections and blood changes as well as hypogammaglobulinemia as most frequent side effect for CAR T cell therapy and CRS, neurotoxicity, infections and infusion reaction for TCE therapy (Fig. 1E).

Similarly, the most important contraindications for CAR T cell therapy were retrieved. Rheumatologists stated heart failure (New York Heart Association, NYHA >/= III), chronic viral infections as well as solid malignant diseases as potential contraindications (Fig. 1C).

## Discussion

This survey on perceptions and expectations regarding TCRT amongst German rheumatologists showed a high overall disposition to engage with this therapy and willingness to refer patients to specialized centers. Yet, only few patients—with refractory, severe diseases, especially connective tissue diseases—were seen as suitable for these therapies. While rheumatologists’ responses regarding selected common adverse events and contraindications were largely consistent with current safety data, self-rated familiarity with TCRTs was moderate.

TCRT is a hot topic in rheumatology, for example the European Alliance of Associations for Rheumatology (EULAR) congress 2025 had 2 sessions, 8 oral presentations and 25 additional posters on this topic. Furthermore, specialized congresses and meetings are hosted by societies of rheumatology and hematology. Many articles, case reports and reviews have been published on TCRTs in rheumatology and lay media such as television shows or newspapers regularly report on this topic in Germany. This intense presence in the rheumatology field might explain the overall good knowledge on TCRT among the participants.

For CAR T cell therapy, most common adverse events like CRS and neurotoxicity were correctly identified, whilst gastrointestinal changes are, according to literature, more prevalent than the suspected infections[18]. Similarly, contraindications towards CAR T cell therapy were correctly stated by most rheumatologists. Heart failure III° or IV° NYHA is a common exclusion criterion for ongoing clinical trials in CAR T cell therapy in autoimmune diseases, as the therapy or its adverse events may display cardiotoxic potential [19, 20]. Additionally, chronic infections or solid malignancies are established as contraindications for clinical trials, as they are for many other immunosuppressive therapies.

In contrast, for TCE therapy, the rarity of neurotoxicity compared to other side effects was less well known. Participants stated neurotoxicity as the second most common adverse events, whilst severe ICANS actually occurs in only 4.3% of the cases [21]. Despite this, other most common adverse events were correctly mentioned.

A possible explanation for this difference between knowledge on CAR T cell and TCE therapy is the fact that CAR T cell therapy was applied and published before TCE therapy in rheumatology [22]: As TCE therapy for rheumatoid arthritis was first published in 2024, only a few months before the survey was initiated, knowledge amongst rheumatologist might not have been as profound.

Interestingly, the knowledge on TCRT did not differ between participants from university (who might possibly offer TCRT at their sites) and non-university hospitals. This underlines the importance of scientific journals, congresses and other media to inform treating physicians, especially for rare diseases and not widely available treatment options.

German rheumatologists appraised especially connective tissue diseases as suitable for TCRT. This might be biased by the so far published case reports, which mainly reported diseases like SLE, myositis, multiple sclerosis or systemic sclerosis [23–25]. Yet, some reports exist on TCRT in rheumatoid arthritis, vasculitis or IgG4-related disease [11, 12, 26, 27]. Overall, these reports addressed patients with therapy refractory disease courses and partly life or organ threatening diseases. Correspondingly, the presented case vignette with refractory disease and severe organ involvement was evaluated as suitable for CAR T cell therapy, while mild disease or initial disease was not seen as appropriate in the other vignettes. These opinions did not differ between participants from academic and non-academic hospitals. Not only the so far published literature, but also the possibilities of therapeutic options differ between the diseases. Whilst in e.g. rheumatoid arthritis yearly new therapies are approved, myositis guidelines state fewer therapeutic options and only intravenous immunoglobulin treatment is officially approved so far [28–30]. Thus, in addition to severe organ involvement, patients with connective tissue diseases might profit even more from these new therapeutic strategies.

Correlating with this aspect, participants from university hospitals thought that more of their patients are suitable for CAR T cell therapy than participants of other hospitals/outpatient clinics (5.95% vs. 3.59%, p 0.016). In Germany, university hospitals mostly have specialized rheumatology inpatient ward with usually severely ill patients, which might explain this difference. Yet, almost all participants (from all workplaces) were willing to refer their suitable patients to specialized centers. However, it is imperative to acknowledge that in Germany, TCRTs are predominantly administered in clinical studies conducted in specialized university hospitals that possess the requisite infrastructure and multidisciplinary expertise. Therefore, the existence of well-established referral pathways is imperative for facilitating access to TCRTs.

The stated main barrier of high treatment costs and limited clinical experience might definitely influence the decision of which patients seem suitable for TCRT. CAR T Cell therapy e.g., has high costs due to manufacturing, but also long hospital stays and required follow-ups in specialized centers [31, 32]. Knowledge about long-term outcomes in rheumatological diseases is very limited. Risks of potential side-effects or malignancies, as they are reported for hematological diseases, were not seen as barriers for this therapy [33, 34]. As previous treatment and underlying disease differ substantially between rheumatological and hematological patients, differences in the prevalence of side effects seem possible.

The limitations of this study are inherent to the survey design. A sampling bias due to the promotion of the survey at the German rheumatology congress is possible. Therefore, more rheumatologists with an academic workplace as well as regular postgraduate clinical training might have been included. Yet, only the number of possibly suitable own patients differed between academic and non-academic workplace. No significant difference between university-hospital and non-university hospital-employed rheumatologists was observed in all other questions (Supplementary Table 1).

A further constraint of the present study is that the survey was conducted exclusively in Germany. Therefore, the potential for its application and generalization in other countries may be constrained by variations in healthcare infrastructure, treatment accessibility, reimbursement mechanisms, and prevailing clinical practices, all of which may influence rheumatologists’ perceptions and expectations regarding TCRTs. Nevertheless, Germany represents an important setting for investigating these perceptions at this early stage of clinical translation. First clinical applications of both CAR T-cell therapy and TCE therapy in rheumatological diseases have been reported in Germany, and Germany remains an important region for clinical research on these emerging treatment approaches [14, 15]. Thus, although the findings should not be considered representative of rheumatologists internationally, this study provides an early assessment of the perspectives of rheumatologists in a country at the forefront of the clinical development and application of TCRTs. Such insights may be particularly relevant during the early adoption phase of novel therapeutic approaches, when expectations, perceived barriers, and clinical decision-making considerations are still evolving.

To facilitate the completion of the survey within a constrained timeframe, the survey's duration and, consequently, the number of questions were restricted. Due to the access via google forms, IP address and survey views were not assessed. Thus, response rate was not assessable and duplicate participation was not preventable, yet we asked participants to only answer one survey per person.

Given that this survey was of exploratory nature, there was neither a formal calculation of the required sample size, nor a calculation to establish the study's power. Despite the 98 respondents providing an initial overview of rheumatologists' perceptions of TCRTs, the limited sample size of the study precludes the possibility of achieving adequate statistical precision and the capacity to conduct sufficiently powered subgroup analyses.

Further studies are necessary to determine the necessity and demand for specialized education on this topic. In addition, the use of established referral pathways for TCRT in specialized centers and in other countries and healthcare systems should be examined to assess the generalisabilty of our findings.

The survey suggests that rheumatologists’ responses regarding selected adverse events and contraindications were largely in line with current safety data. However, self-rated overall knowledge of TCRTs was only moderate. At the same time, the results show considerable interest in these therapies among rheumatologists, while high treatment costs, limited clinical experience, and the restricted patient population were identified as key barriers to their wider use.

## Conclusion

In conclusion, our study demonstrates that the participating German rheumatologists recognize the therapeutic potential of T-cell redirecting therapies (TCRTs), especially for patients with highly active systemic diseases and a history of extensive immunosuppressive treatment. Our study showed that most participants are willing to refer their own patients to specialized centers for TCRT and knowledge about these therapies appeared moderate. However, the broader application of TCRTs in autoimmune diseases is currently impeded by high treatment costs and the need for more robust clinical data. Future research should focus on addressing these barriers to facilitate wider access and utilization of TCRTs in clinical practice.

## Supplementary Information

Below is the link to the electronic supplementary material.

Supplementary file1 (DOCX 48 KB)

## Data Availability

The data that support the findings of this study are available from the corresponding author upon reasonable request.

## Abbreviations

TCRT: T-cell redirecting therapy
TCE: T-cell engager
SLE: Systemic lupus erythematosus
ICANS: Immune effector cell-associated neurotoxicity
CRS: Cytokine release syndrome
ANCA: Anti-neutrophil cytoplasmic antibody
BCMA: B-cell maturation antigen
NYHA: New york heart association
EULAR: European alliance of associations for rheumatology
