# Supplementary material for: Rheumatologists’ expectations and perceptions of T-cell redirecting therapies: results from a cross-sectional survey
Source: Rheumatol Int. 2026 Sep 26;46(10):264. doi: 10.1007/s00296-026-06300-3 (PMC13615921; doi:10.1007/s00296-026-06300-3)
Supplement: Supplementary file 1 [file 296_2026_6300_MOESM1_ESM.docx]

**Rheumatologists’ Expectations and Perceptions of T-Cell Redirecting Therapies: Results from a Cross-Sectional Survey**

Anna Meinecke^1,2,3^, Johannes Knitza^3,4^, Thorben Witte^3,5^, Judith Rademacher^3,6^, Felix Müller^3,7^, Christina Düsing^3,8^, Marco Zeeck^3,9^, Ricardo Grieshaber-Bouyer^3,10, 11^, Melanie Hagen^3,10, 11^, Isabell Haase^3,12^*, Marie-Therese Holzer^3,12^* on behalf of the Working Group Young Rheumatologists of the German society for Rheumatology and Immunology

1 Department of Rheumatology and Immunology, Hannover Medical School, Hannover, Germany.2 PRACTIS Clinician Scientist Program, Dean’s Office for Academic Career Development, Hannover Medical School, Hannover, Germany

3 Working Group Young Rheumatology, German Society for Rheumatology, Berlin, Germany
4 Institute for Digital Medicine, Philipps University Marburg, Marburg, Germany. 5 Department of Rheumatology and Clinical Immunology, Charité - Universitätsmedizin Berlin, Berlin, Germany.
6 Department of Gastroenterology, Infectiology and Rheumatology (including Nutrition Medicine), Charite Universitätsmedizin Berlin, Berlin, Germany.
7 Division of Rheumatology & Clinical Immunology, Department of Medicine 3, University Hospital Augsburg, Augsburg, Germany

8 Department of Rheumatology, University Hospital Düsseldorf, Medical Faculty of Heinrich-Heine University; Düsseldorf, Germany
9 Rheuma Zentrum Lübeck, Lübeck, Germany
10 Deutsches Zentrum Immuntherapie (DZI) Friedrich-Alexander Universität (FAU) Erlangen-Nürnberg, Erlangen, Germany

11 Department of Internal Medicine 3 – Rheumatology and Immunology, Friedrich-Alexander Universität (FAU) Erlangen-Nürnberg and Universitätsklinikum Erlangen, Erlangen, Germany
12 Division of Rheumatology and Systemic Inflammatory Diseases, III. Department of Medicine, University Medical Center Hamburg-Eppendorf, Hamburg, Germany.

*shared last authorship

**Supplementary information**

**Supplementary table 1:**

|  |  | University hospital (n=44) | Non-University hospital (n=51) | Mann Whitney Test |
| --- | --- | --- | --- | --- |
| Patients suitable for TCRTs | mean [95%CI] | 5.95% [3.39%;8.51%] | 3.59% [2.07%;5.12%] | p=0.016 |
| Knowledge regarding TCRTs | mean | 5.5/10 | 4.41/10 | p=0.072 |
|  |  |  |  |  |
| case vignettes |  | **University hospital (n=44)** | **Non-University hospital (n=50-51)** | **Fisher's exact test** |
| Case 1 | Agree to send to TCRTs | 34/44 (77.3%) | 33/50 (66.0%) | p=0.020 |
| Case 2 | Agree to send to TCRTs | 3/44 (6.8%) | 1/50 (2%) | p=0.666 |
| Case 3 | Agree to send to TCRTs | 5/44 (11.4%) | 6/51 (11.7%) | p=0.226 |

**Translated survey (from original German language):**

1. How old are you?

_________________

2. Which gender do you identify with? Please select only one option.

- Female
- Male
- Other
- Not specified

3. What is your job title? Select only one option.

- Rheumatologist in training
- Board-certified specialist
- Senior Physician
- Chief Physician
- Not specified

4. What is your employment status? Select only one option.

- Employed at a practice/medical care center
- Private practice
- Municipal hospital
- Tertiary care facility
- University hospital
- Specialized rheumatological clinic
- No information provided

# 5. What percentage of your patients do you think would be eligible for cell-specific therapy (CAR-T cell therapy or T cell engager therapy)? Please provide a number out of 100.

_____________________

6. Which disease groups do these patients belong to? (Multiple answers allowed) Select all that apply.

- RA/SpA
- Systemic Sclerosis Non-ANCA vasculitides
- Autoinflammatory diseases
- Inflammatory myositis
- Systemic lupus erythematosus without lupus nephritis
- Systemic lupus erythematosus with lupus nephritis
- Primary Sjögren’s syndrome
- Overlap connective tissue disease
- ANCA vasculitides
- IgG4-associated diseases
- Other: _______________
- Not applicable, as none of my patients would qualify
- No information provided

7. Would you refer eligible patients to a specialized center for treatment and continue to provide follow-up care? Select only one option.

- Yes
- No
- No response
- Question not applicable, as there are no suitable patients

8. If no, what are the reasons?

___________________________

9. How would you rate your own knowledge of CAR-T cell therapy or T cell engager therapy?

0	1	2	3	4	5	6	7	8	9	10

None	optimal prior knowledge

10. What are the 4 most common side effects of CAR-T cell therapy? (Multiple answers allowed) Select all that apply.
    - Cytokine release syndrome (CRS)
    - Immune Effector Cell-Associated Neurotoxicity Syndrome (ICANS)
    - Infusion reaction
    - Cardiac side effects
    - Hemophagocytic lymphohistiocytosis
    - Infections
    - pulmonary fibrosis
    - Risk of malignancy
    - Changes in Blood Count
    - Hypogammaglobulinemia
    - Immunologically Induced Autoimmune Disease
    - Gastrointestinal Side Effects
    - No response

- Other: _______________

11. What are the 4 most common side effects of T-cell engager therapy? (Multiple answers allowed) Select all that apply.
    - Cytokine release syndrome (CRS)
    - Immune Effector Cell-Associated Neurotoxicity Syndrome (ICANS)
    - Infusion reaction
    - Cardiac side effects
    - Hemophagocytic lymphohistiocytosis
    - Infections
    - pulmonary fibrosis
    - Risk of malignancy
    - Changes in Blood Count
    - Hypogammaglobulinemia
    - Immunologically Induced Autoimmune Disease
    - Gastrointestinal Side Effects
    - No response

- Other: _______________

12. Which of your patients’ comorbidities do you consider to be contraindications for CAR-T cell therapy in rheumatology patients? (Multiple answers allowed) Select all that apply.

- Heart failure NYHA ≤ II
- Heart failure NYHA ≥ III
- Underlying neurological condition (e.g., stroke, MS)
- Pulmonary fibrosis
- Chronic viral infection (HIV, hepatitis)
- CMV positivity
- Solid malignancy
- Uncontrolled underlying disease
- Immunosuppression cannot be paused
- Other: _______________
- No response

13. What do you consider to be “barriers” to immune cell therapies (T-cell redirecting therapy)? (Multiple answers allowed) Select all that apply.

- High treatment costs
- Suitable for only a few patients
- Long follow-up period required by law
- Currently little clinical experience available
- Not sufficiently researched to date
- Patients too ill for therapy
- Antibody therapies are more effective
- Cannot be performed on an outpatient basis
- range of dangerous side effects
- risk of malignancy
- Other: _______________
- No response

**Case vignettes**

14. Patient, 58 years old, with progressive systemic sclerosis and progressive pulmonary involvement (vital capacity 45%, DLCO 40%), tendon friction, Scl70+. Previous treatments: cyclophosphamide.

Would you administer CAR-T cells in this case? Select only one oval.

- Yes
- No
- Don’t know

15. Patient, 24 years old, with systemic lupus erythematosus involving the skin and joints, as well as alopecia (CLASI score 16 points). Previous treatments: prednisolone, hydroxychloroquine. Would you administer CAR-T cells in this case? Select only one oval.

- Yes
- No
- Don’t know

16. Patient, 45 years old, with acute granulomatous polyangiitis and pulmonary hemorrhage, intubated in the intensive care unit. Previous treatment: prednisolone.

Would you administer CAR-T cells in this case? Select only one oval.

- Yes
- No
- Don’t know
